# Nucleoplasmin-like domain of FKBP39 from *Drosophila melanogaster* forms a tetramer with partly disordered tentacle-like C-terminal segments

**DOI:** 10.1038/srep40405

**Published:** 2017-01-11

**Authors:** Małgorzata Kozłowska, Aneta Tarczewska, Michał Jakób, Dominika Bystranowska, Michał Taube, Maciej Kozak, Mariusz Czarnocki-Cieciura, Andrzej Dziembowski, Marek Orłowski, Katarzyna Tkocz, Andrzej Ożyhar

**Affiliations:** 1 Department of Biochemistry, Faculty of Chemistry, Wrocław University of Science and Technology, Wybrzeże Wyspiańskiego 27, 50-370 Wrocław, Poland; 2 Department of Macromolecular Physics, Faculty of Physics, Adam Mickiewicz University, Umultowska 85, 61-614 Poznań, Poland; 3 International Institute of Molecular and Cell Biology in Warsaw, 02-109 Warszawa, Poland; 4 Institute of Biochemistry and Biophysics, Polish Academy of Sciences, 02-106 Warszawa, Poland; 5 Department of Genetics and Biotechnology, Faculty of Biology, University of Warsaw, 02-106 Warszawa, Poland

## Abstract

Nucleoplasmins are a nuclear chaperone family defined by the presence of a highly conserved N-terminal core domain. X-ray crystallographic studies of isolated nucleoplasmin core domains revealed a β-propeller structure consisting of a set of five monomers that together form a stable pentamer. Recent studies on isolated N-terminal domains from *Drosophila* 39-kDa FK506-binding protein (FKBP39) and from other chromatin-associated proteins showed analogous, nucleoplasmin-like (NPL) pentameric structures. Here, we report that the NPL domain of the full-length FKBP39 does not form pentameric complexes. Multi-angle light scattering (MALS) and sedimentation equilibrium ultracentrifugation (SE AUC) analyses of the molecular mass of the full-length protein indicated that FKBP39 forms homotetrameric complexes. Molecular models reconstructed from small-angle X-ray scattering (SAXS) revealed that the NPL domain forms a stable, tetrameric core and that FK506-binding domains are linked to it by intrinsically disordered, flexible chains that form tentacle-like segments. Analyses of full-length FKBP39 and its isolated NPL domain suggested that the distal regions of the polypeptide chain influence and determine the quaternary conformation of the nucleoplasmin-like protein. These results provide new insights regarding the conserved structure of nucleoplasmin core domains and provide a potential explanation for the importance of the tetrameric structural organization of full-length nucleoplasmins.

Insect development and growth are controlled by two major lipophilic hormones, 20-hydroxyecdysone (20E) and juvenile hormone (JH)^1^. In contrast to 20E-dependent signalling, which is thoroughly characterized, the mechanism of action of JH remains largely unknown^2^, and the cross-talk between these two signalling pathways remains a puzzle. Interestingly, Li *et al*.^3^ suggested that two proteins, the 39-kDa FK506-binding protein (FKBP39) and the 21-kDa calponin-like protein (Chd64), are key components of a dynamic, multiprotein complex that crosslinks these two hormonal signalling pathways. This putative complex also includes ecdysone receptor (EcR), *Ultraspiracle* (Usp) and *methoprene-tolerant* (Met) protein and can bind to the juvenile hormone response element (JHRE)^3^. However, the molecular basis of the interactions between FKBP39, Chd64 and the other components of the multiprotein complex is not understood. Recently, Kozlowska *et al*.^4^ showed that the structure of Chd64 possesses a dual nature, consisting of a globular domain flanked by intrinsically disordered tails. This intrinsic disorder (ID) originates from a biased amino acid composition, low sequence complexity and a lack of bulky hydrophobic residues, which prevent spontaneous folding into stable secondary and tertiary conformations. Intrinsically disordered proteins (IDPs) and intrinsically disordered regions (IDRs) are pliable and dynamic structures that exist in various conformations^5^. Despite the lack of a stable, well-defined structure, IDPs/IDRs are fully functional and very often multifunctional. Their flexible conformations allow IDPs/IDRs to expose short linear peptide motifs, which are responsible for multiple interactions. Moreover, their pliable structures can easily adapt to different binding partners. Thus, one IDP/IDR often binds a wide range of proteins^6^,^7^. For these reasons, the presence of disorderliness at the N- and C-termini of Chd64 may lay a foundation for its biological function because the labile, dynamic and highly adjustable nature of disordered regions could enable interaction with various partners^4^. The second key component of the putative multiprotein complex that crosslinks the 20E and JH signalling pathways is FKBP39. This protein belongs to the immunophilins, a group of immunosuppressive drug-binding proteins^8^,^9^. Ligands bind to the hydrophobic FK506-binding domain (FKBD)^10^, which is well-preserved evolutionarily^11^. Many FKBDs possess catalytic, peptidyl-prolyl *cis-trans* isomerase (PPIase) activity. Prolyl bond conversion, although spontaneous, is a rate-limiting step in the folding of nascent protein. PPIases can also induce conformational changes in mature proteins^12^. Therefore, FKBPs perform a number of important regulatory functions in different cellular compartments of all taxonomic groups of living organisms. For example, FKBP39 from *Schizosaccharomyces pombe* and Fpr4 from *Saccharomyces cerevisiae* have been extensively studied because of their role in the regulation of chromatin structure^13^,^14^,^15^. The former supercoils plasmid DNA in a histone-dependent manner, suggesting that it promotes nucleosome assembly^13^. Xiao *et al*.^14^ showed that nucleosome assembly is executed by acidic regions of FKBP39, prompting similar actions by Fpr4 and the classical histone chaperone NAP-1^14^. Additionally, Kuzuhara and Horikoshi^13^ showed that both proteins are enriched in rDNA loci in the nucleolus and are involved in their silencing^13^. Interactome analysis revealed a physical association of Fpr4 with ribosome biogenesis factors^16^,^17^. These findings indicate that FKBP39-related proteins perform important and complex but still vague nuclear functions that require further exploration. Additional structural studies are also needed. Very recently, structural studies of isolated N-terminal domain from *Drosophila* FKBP39 revealed high structural homology to a core domain of nucleoplasmin, and its functional involvement has been suggested^18^. Nucleoplasmins play a role in chromatin organization by providing a long-lasting storage platform for histones^19^. The crystal structures of the isolated nucleoplasmin core domains from different species revealed oligomeric structures in which pentamers and decamers were formed by packing two pentamers on top of each other. Decameric structure is assumed to be involved in the binding of histone complexes^20^.

In this paper, we present a detailed structural analysis of the full-length FKBP39 from *Drosophila melanogaster*, a two-domain protein with a long, highly charged linker. Our data indicated an unusual quaternary organization of FKBP39 that distinguishes this protein from other family members. Molecular mass (Mm) analyses indicated that the full-length protein forms tetramers. A high-resolution small-angle X-ray scattering (SAXS) model demonstrated that FKBP39 monomers fit together via the N-terminal nucleoplasmin-like (NPL) domain, forming a stable core. C-terminal FKBDs are linked to this core by intrinsically disordered, highly flexible chains, resulting in tentacle-like segments. We also describe the subcellular distribution of FKBP39 in living cells. The importance of this unique structural organization and the subcellular localization of FKBP39 in hormonal signal transduction and chromatin remodelling are discussed.

## Results

### *Drosophila* FKBP39 is partly disordered

**Figure 1:**
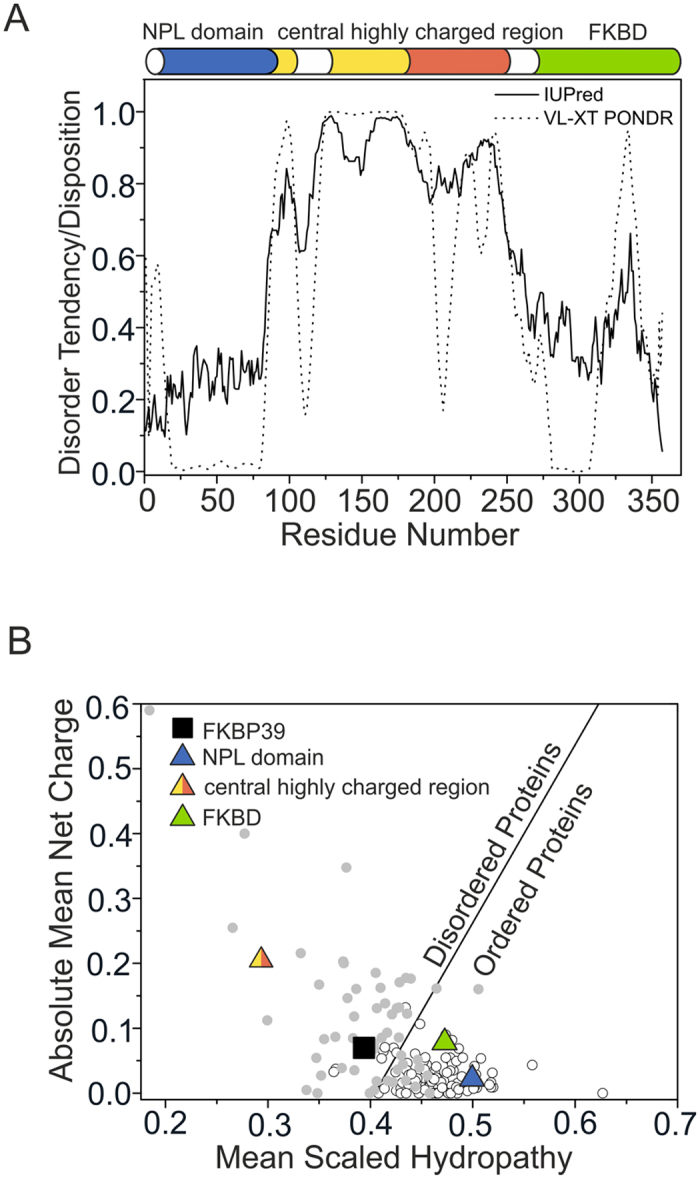
Analysis of FKBP39 disorderliness. (**A**) Search for conserved domains and disordered regions of the full-length FKBP39. The top of the panel represents the schematic domain structure of FKBP39 obtained by Pfam^67^. N-terminal NPL is presented in blue; the central highly charged region consists of acidic (yellow) and basic stretches (red), and the C-terminal FKBD is shown in green. The bottom of the panel presents a graphical prediction of the degree of disorder in FKBP39 calculated from its amino acid sequence using IUPred (solid lines) and PONDR-VLXT (dotted lines). A disorder tendency/disposition score above 0.5 indicates a high probability of disorder. (**B**) The Uversky plot^53^,^54^. Full-length FKBP39 (black square) is located in an ambiguous zone occupied by both types of proteins: globular (white dots) and disordered (grey dots). N-terminal NPL (1–86) and C-terminal FKBD (257–357), represented by the blue and green triangles, respectively, are located among the ordered proteins, whereas the central highly charged region (87–256), represented by a yellow/red triangle, is located in a zone occupied by IDPs.

FKBP39 has been shown to be involved in the cross-talk of hormonal signalling pathways and, therefore, plays an important regulatory role in the development and growth of *D. melanogaster.* Li *et al*.^3^ showed that the cross-talk of the pathways that govern the proper passage from one developmental checkpoint to the next is fulfilled by a dynamic, multiprotein complex. FKBP39 and Chd64 were shown to modulate and recruit that complex to appropriate regulatory elements within the promoters of respective genes in response to existing signals. However, neither the mechanism nor the molecular bases of the interaction with various putative partners have been elucidated^3^. In addition, nothing is known about the structure of the full-length FKBP39. To obtain insight into its molecular properties and better understand FKBP39 molecular function, we performed complex biochemical and biophysical analyses. Because a flexible and adaptive disordered conformation is advantageous for proteins involved in the transmission of biological processes^21^, we began by searching for ID. For the substantive search, we chose two web-based predictors that act on different assumptions^22^,^23^. As presented in Fig. 1A, at the FKBP39 N-terminus, the first 86 amino acid residues have a low disorder tendency of approximately 0.25, suggesting the existence of an ordered structure. This prediction is in agreement with the results of a search for conserved domains by Phyre2 software (data not shown) and a recent nuclear magnetic resonance (NMR) structural study of the N-terminal fragment of FKBP39, which also indicated an ordered structure^18^. Similarly, the C-terminal fragment of FKBP39 between 257 and 357 amino acid residues, excluding a short fragment between residues 321 and 339, did not exhibit a disordered tendency, with a score below 0.5. A Phyre2 search and sequence comparison with FKBP39 homologs revealed a putative FKBD domain on the C terminus of the protein (not shown). The FKBD-fold is well preserved in all known family members, including noncanonical FKBPs, which do not have catalytic activity. β-sheets constitute the majority of the secondary structure and form hydrophobic grooves responsible for binding lipophilic ligands and proline *cis-to-trans* isomerization^24^. In contrast to the terminal fragments of FKBP39, the central, highly charged region (residues 86–256) was predicted to have a high disorder tendency. In that fragment, the IUPred score was 0.6–1.0. PONDR-VLXT identified two shorter fragments at residues 106–117 and 201–212 where the disorder tendency was greatly decreased, possibly indicating the presence of some residual secondary structures or molecular recognition elements (MoREs) that undergo folding upon binding^25^. To further investigate the conformation of FKBP39, we analysed selected fragments using a method introduced by Uversky^5^, in which the mean net charge and mean hydropathy are calculated. These values are plotted in a scaled charge-hydropathy space where a linear boundary separates disordered and globular proteins. As presented in Fig. 1B, the full-length FKBP39 fell into the ambiguous zone occupied by both disordered and globular proteins. In contrast, NPL and FKBD domains fell into a group of globular proteins, whereas the central, highly charged region was located exclusively in the zone of IDPs.

**Figure 2:**
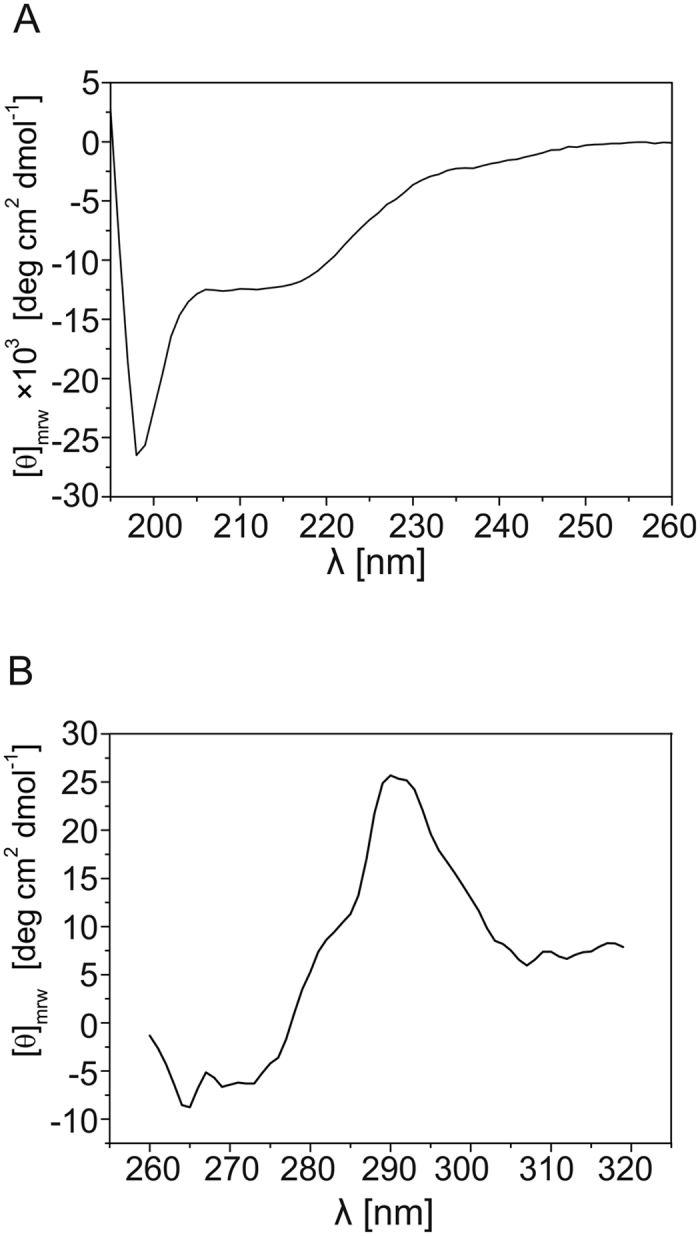
CD spectra of FKBP39. (**A**) Far-UV spectrum. Five different measurements of 10 μM (40 μg/ml) protein were taken. The averaged spectrum of FKBP39 displays two prominent negative maxima. The first, which is at approximately 195 nm, indicates an extensive disordered conformation, whereas the second, which is at 218 nm, is characteristic of an ordered β-sheet structure. (**B**) The near-UV spectrum of FKBP39. The presented spectrum is an average of five different measurements recorded for a 25 μM concentration of protein. The major peak around 292 nm represents the band for Trp, whereas the peak at 278 is due to Tyr residues. All spectra were recorded in buffer C at room temperature.

To perform *in vitro* biochemical and biophysical analyses of the FKBP39 structure, we developed and optimized an overexpression and purification protocol. The final sample containing FKBP39 was at least 95% pure based on densitometry analyses of polyacrylamide Coomassie Brilliant Blue R-250-stained gels (data not shown). To confirm the identity of the protein, the Mm value was determined via electrospray ionization mass spectrometry (ESI-MS). The experimental value of 40,696.6 ± 2 Da was similar to the theoretical value of 40,826.1 Da for the full-length protein in the absence of the first N-terminal methionine (129.5 Da). Cleavage of the N-terminal methionine is well documented for recombinant proteins expressed in prokaryotic systems^26^. The apparent Mm was also determined using sodium dodecyl sulfate polyacrylamide gel electrophoresis (SDS-PAGE). According to the migration pattern of the unstained protein molecular marker (Fermentas), we found that FKBP39 migrates as its globular equivalent of 48.8 kDa (data not shown). The 20% difference between the theoretical and experimental Mm values is attributable to the specific amino acid compositions of IDPs/IDRs, which bind less SDS^27^. To validate the *in silico* analysis, which indicated a disordered conformation in FKBP39, we used circular dichroism (CD) spectroscopy. The far-UV CD spectrum (Fig. 2A) includes two deep negative maxima: one below 200 nm, which is characteristic of disordered conformations, and one at 218 nm, indicating a high content of ordered secondary structure^28^. CDPro deconvolution software was utilized to perform a more detailed numerical analysis of the impact of the secondary structure. CDSSTR, SELCON3 and CONTINLL algorithms were chosen, and the results obtained using the IBasis 4 database are summarized in Table 1. The analysis revealed that 39.3 ± 0.5% of the polypeptide chain length was in a regular or distorted β-structure. The high β-structure content is in agreement with the fact that both FKBD and NPL domains mostly consist of β-structures, with α-helices comprising only 3.1 ± 0.3% of the protein structure. Thus, 34.6 ± 0.2% of the amino acid residues are in a disordered conformation. The CD results are consistent with the *in silico* analysis, which predicted that the central, highly charged region of the FKBP39 polypeptide chain exists in a disordered conformation. Additionally, we analysed the contribution of the aromatic amino acids using near-UV CD (Fig. 2B). The FKBP39 sequence contains two Trp, nine Tyr, and nine Phe residues. The obtained spectrum is well developed, with a prominent positive band at 292 nm that is unique to Trp residues (one located within NPL and one in FKBD) and a minor signal at 278 nm that can be ascribed to the tyrosyl side chain. The shape of the curve and the ellipticity values in the near-UV region indicate the presence of a well-developed tertiary structure.

### FKBP39 forms homotetramers

**Figure 3:**
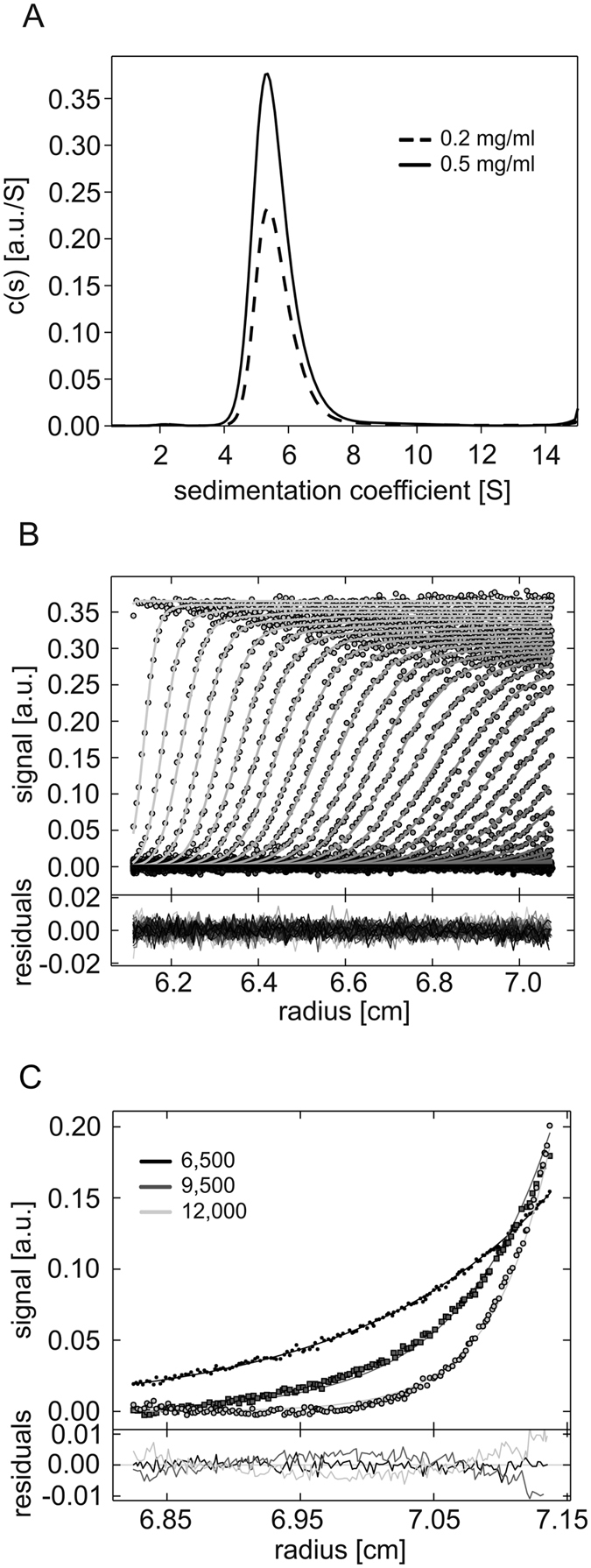
AUC of FKBP39. (**A**) SV-AUC analysis of FKBP39. c(s) distribution derived via the continuous c(s) distribution model of SEDFIT, standardized to water at 20 °C. The SV absorbance at 280 nm was determined at a concentration of 0.2 (dashed line) and 0.5 (solid lines) mg/ml. A single peak is visible with an s_20,w_ of 6.07 S, which corresponds to an R_s_ of 6.34 nm and an *f/f*_*0*_ of 1.75. (**B**) Statistical analysis. Superposition of experimental data (depicted as circles) and fitted (continuous line) SV profiles corrected for systematic noises. The data are well fitted with rmsd of 0.003. The lower panel shows the superposition of the differences between the experimental and fitted curves. (**C**) A representative result of SE-AUC experiments performed at 4 °C with 0.44 mg/ml FKBP39. The samples were spun in multisided mode at 6,500 (dots), 9,500 (squares), and 12,000 rpm (open circles). All SE experiments were globally fitted using SEDPHAT software, resulting in a good statistical analysis (residuals inset) and well-fitted data (continuous lines).

**Figure 4:**
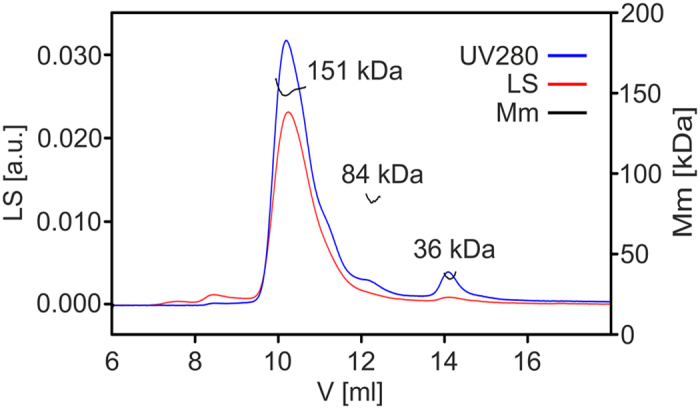
Molecular mass determination by SEC-MALS. Analysis of the oligomeric state of the FKBP39 protein by SEC-MALS. Average Mm values as measured by MALS are given by the selected peaks. The experimental Mm for the FKBP39 monomer as determined by ESI MS is 40.696 kDa. The blue line corresponds to the absorbance measured at 280 nm, the red line presents light scattering (LS) at 90 degrees, and the three separated black lines correspond to the average Mm values of FKBP39 in different oligomeric states.

Analytical size exclusion chromatography (SEC, see Fig. S1 and Table S1) and sedimentation velocity analytical ultracentrifugation (SV-AUC) experiments were conducted to estimate the size distribution of FKBP39 and its shape-related parameters. The cumulative spectra from SV-AUC experiment were analysed using the continuous size distribution model^29^. The results indicate a single distribution peak containing 96.9% of the signal (Fig. 3A,B), suggesting the presence of only one species in the solution with a sedimentation coefficients (s_20,w_) of 6.07 S, which corresponds to a Stokes radius (R_s_) of 6.34 nm. The species contributing to the distribution peak result in a frictional ratio (*f/f*_*0*_) of 1.75, suggesting an elongated oval/rod-shaped protein^29^ with an estimated Mm of 160.7 kDa, which corresponds to a tetrameric arrangement of FKBP39 (Table 2). To elucidate the protein’s exact Mm, we first used data obtained during sedimentation equilibrium AUC (SE-AUC), which is a first-principles method to determine the Mm of macromolecules. The data collected with three different protein concentrations at three centrifugation speeds were analysed globally in SEDPHAT using a single-species model and resulted in a good fit with rmsd of 0.003 (Fig. 3C). As presented in Table 2, the Mm obtained for FKBP39 was 156.8 kDa, which was in good agreement with the theoretical mass of the FKBP39 tetramer (Mm 162.780 kDa). Analysing the SAXS data (see below) provided additional evidence regarding the oligomerization state of FKBP39. The average Mm of FKBP39 obtained from the SAXS data sets using the MoW program^30^ was 158 ± 14 kDa. This value corresponds well to the theoretical mass of the FKBP39 tetramer. Finally, the Mm of oligomeric FKBP39 was determined using another independent method, SEC coupled with Multiangle Light Scattering (MALS). FKBP39 was chromatographed on a Superdex 200 Increase 10/300 column. As presented in Fig. 4, most of the protein eluted in a single peak with an elution volume of 10.2 ml. According to MALS, the average Mm of the proteins that eluted in this peak was 151.0 kDa, which corresponds to a tetramer. Two other forms with elution volumes of 12.1 ml and 14.1 ml and average Mm values of 84 kDa and 36 kDa, respectively, correspond to a dimer (Mm theoret. 81.4 kDa) and a monomer (Mm theoret. 40.7 kDa). To further investigate the size distribution and oligomeric state of recombinant FKBP39, dynamic light scattering (DLS) analysis was carried out. The intensity size distribution plots revealed a unique population with a polydispersity (%Pd) value of approximately 12% (Fig. S2). In terms of protein analysis, a %Pd value of less than 20% indicates that the sample is monodisperse^31^. Taken together, these results clearly show that FKBP39 preferentially forms homotetrameric structures.

### FKBP39 forms dynamic and partly disordered tetramers with tentacle-like C-termini

**Figure 5:**
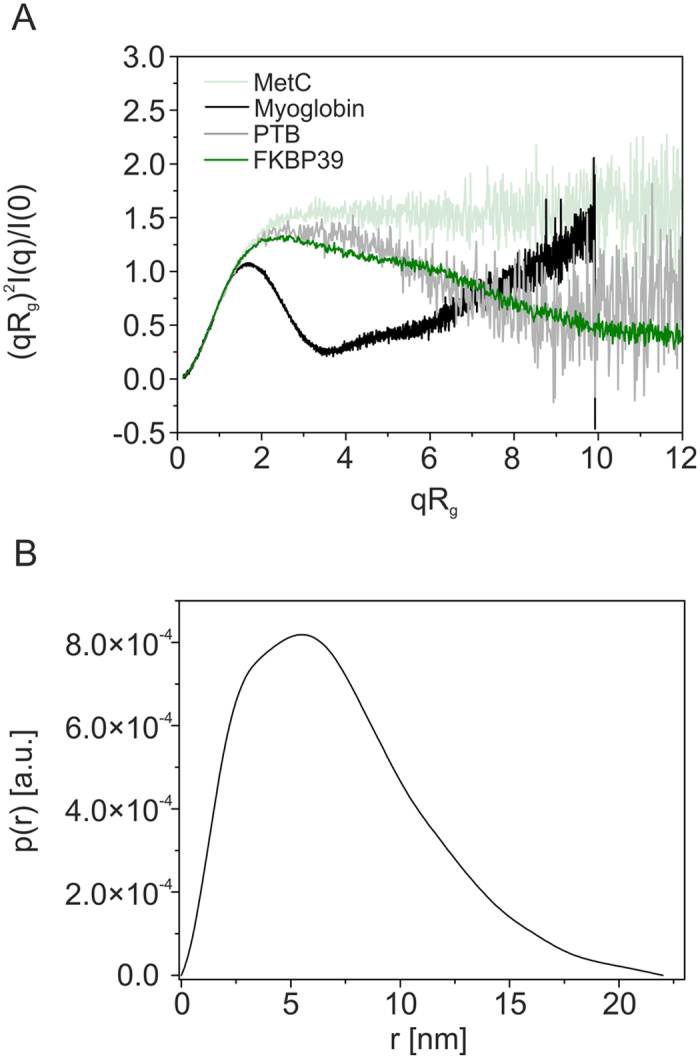
SAXS analysis of the FKBP39 protein in solution. (**A**) Kratky plot of the solution scattering data. The scattering SAXS curve of FKBP39 (dark green) shows a broad, asymmetric peak and a plateau, which are typical of partially disordered proteins. Exemplary scattering curve of coil-like C-terminus of Methoprene-tolerant (MetC; id: SASDBY5) is shown in light green, partly disordered Polypirimidine Tract Binding Protein (PTB; id: SASDAR4) is shown in grey, and globular myoglobin (id: SASDAH2) is shown in black. (**B**) Pair distance distribution function p(r) characterizing the FKBP39 tetramer. The shape of the p(r) function is characteristic for elongated molecules.

**Figure 6:**
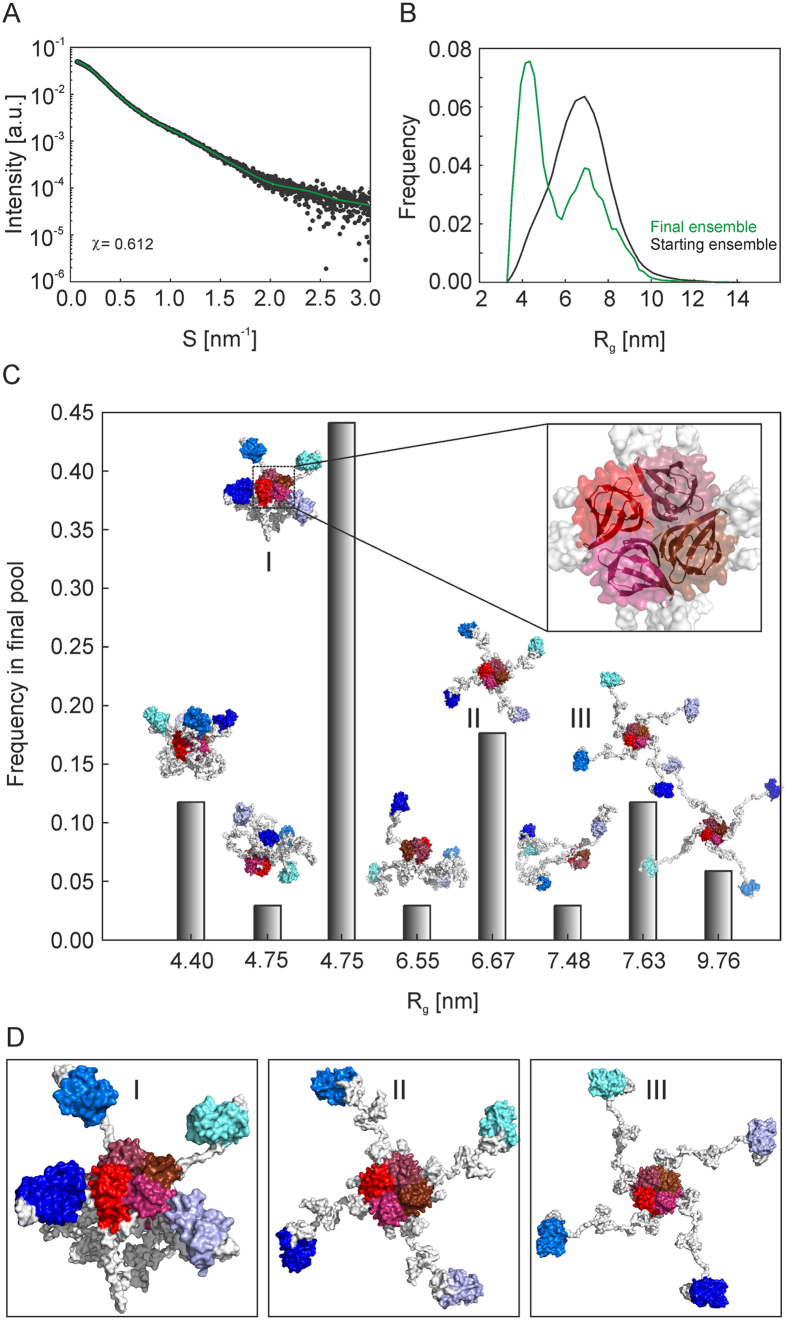
Modelling of the FKBP39 molecule. (**A**) The fit (green) of the best profiles determined by the EOM modelling to SAXS data (black). (**B**) Distribution of R_g_ profile of the starting random population of models (black) and profiles of the final optimized ensemble (green). (**C**) The population of EOM models potentially adopted by the FKBP39 tetramer is presented as a distribution of frequency versus R_g_ values. The inset shows the superposition of the NMR structure of the NPL monomers of the Fpr4 protein (PDB id: 4BF8) onto the tetrameric NPL domain of FKBP39. (**D**) Representative most frequent models presenting the flexible and dynamic nature of the FKBP39 tetramer.

As demonstrated above, FKBP39 appears to be an IDP that forms stable tetramers in solution. To obtain deeper insight into the structure of tetramer, we used SAXS, which can be applied to structural studies of IDPs. An exemplary SAXS scattering curve is presented in Fig. 5A as a Kratky plot. Compared to the bell-shaped Kratky plots exclusively observed for globular proteins, the shape of the plot obtained for FKBP39 is characteristic of partially disordered proteins^32^, confirming our other observations. In the initial step of SAXS data analysis, the basic structural parameters (radius of gyration, R_g_ and maximum intramolecular distance, D_max_) were estimated. The function p(r), which reflects the frequencies of the distances r within a protein molecule, was calculated for FKBP39 by indirect Fourier transformation of experimental solution scattering data. An exemplary pair distribution (p(r)) function is presented in Fig. 5B. The FKBP39 tetramer is characterized by a D_max_ of 22.5 nm and an R_g_ of 5.7 nm, and the profile of the p(r) function indicated that FKBP39 had an elongated shape^33^. Based on the CD data, we assumed that a substantial part of the FKBP39 subunit is ordered and that, therefore, its overall conformation can be restored from the SAXS data^34^. The qualitative information from the Kratky plot (Fig. 5A) indicates that FKBP39 molecules in solution are only partially folded. We realized that the disordered portion of the FKBP39 molecule can result in the formation of variety of different conformations in solution. The Mm range determined from the SAXS data, the susceptibility of the structural changes induced by synchrotron radiation, and a recently published paper reporting that an isolated N-terminal fragment of *Drosophila* FKBP39 can be defined as a homopentameric NPL structure^18^ inspired us to perform a deep and detailed analysis of the SAXS data. The observed Mm range of FKBP39 revealed quite clearly that the dominant oligomeric form in solution is a tetramer (Table 2). However, because of the conformational flexibility of at least partly intrinsically disordered FKBP39 molecules, we cannot ignore the possibility of an alternative oligomerization state. To develop reliable models of FKBP39 in solution, the tetramer of the NPL domain was generated using the SymmDock server^35^ and the NMR structure of the isolated N-terminal domain of *D. melanogaster* FKBP39 (pdb id: 4CA9). The atomic model of the C-terminal FKBD (residues 196–353) was downloaded from the SWISS-MODEL Repository^36^. This structure was modelled on the basis of the FKBD domain structure of the Fpr4 protein (51.33% identity, PDB id: 4BF8), which also possesses an N-terminal NPL domain and a C-terminal FKBD domain. In the next step, these models were used for modelling the FKBP39 structure using the Ensemble Optimization Method (EOM). The components of the FKBP39 chain that were not predicted by the selected atomic models were represented as dummy-atom residues^37^,^38^. The population of ten thousand independent models of FKBP39 was generated using the Ranch program in the ATSAS package. Half of the population of models was generated without defined local symmetry (p1), and the other half possessed local p4 symmetry. The EOM models were then processed using the PD2 ca2main program to generate models in which the residues were replaced with a C-alpha backbone^39^. The models prepared this way were used as a starting pool in the Gajoe program (version 2.0)^38^ in the ATSAS package; this program selects a pool of models that best fits the experimental data. The fit of the best profiles determined by the EOM modelling to the SAXS data is presented in Fig. 6A. The histogram of R_g_ values characterizing the initial pool of models and final ensemble of models after minimization are presented in Fig. 6B. As a result of EOM modelling, we received the illustrations of the type of conformations potentially adopted by the FKBP39 conformers, which are shown in Fig. 6C. These different conformations contributed to the final pool of EOM models with varying frequency (3–44%). A comparison of the most frequently selected models of the FKBP39 tetramer, which were characterized by R_g_ values of 4.75, 6.67 and 7.63 nm, can be observed in Fig. 6D. The stiffened core of the FKBP39 molecule formed by NPL domains is located in the centre of a tetramer, from which the structured FKBD domains connected by flexible tentacle-like linkers propagate.

### FKBP39 is a nucleolar protein

**Figure 7:**
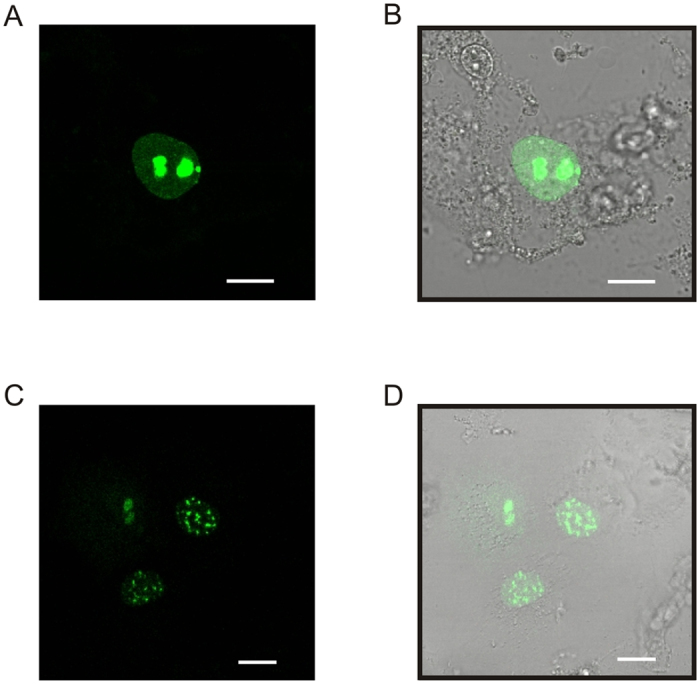
Subcellular distribution of FKBP39 tagged with YFP in COS-7 cells. (**A,C**) Typical cellular localization of YFP-FKBP39 in COS-7 cells observed by confocal imaging recorded 20 h after transfection. (**B,D**) Non-confocal transmitted light images of the same fields of view merged with the images presented in panels (**A**) and (**C**). The bar represents 10 μm.

Due to the extraordinary structural characteristics of recombinant FKBP39, we performed a subcellular localization analysis of a yellow fluorescent protein-FKBP39 fusion construct (YFP-FKBP39) in a heterologous cell culture system (Fig. 7). COS-7 cells were transfected with constructed plasmid encoding the cDNA of FKBP39. The distribution of YFP-FKBP39 was analysed by confocal fluorescence microscopy. We observed that the distribution of YFP-FKBP39 was almost exclusively nucleolar in all the cells analysed (Fig. 7A,B). Interestingly, in a minority of cells, YFP-FKBP39 was localized in the nuclear area in small clusters or visible dots in a “cuckoo egg-like” pattern (Fig. 7C,D). This pattern remained constant for a long time during confocal analysis, and it was reproducible after many transfection experiments.

## Discussion

Our detailed structural analysis of the full-length FKBP39 from *D. melanogaster* revealed that this protein is partly disordered and possesses a unique quaternary structure that may be significant for its regulatory activities. Importantly, a range of independent techniques implemented in this study revealed that the Mm of the FKBP39 oligomer corresponds to the theoretical weight of the tetramer. Therefore, our findings stand in opposition to those of a recent structural study on an isolated N-terminal NPL domain^18^ from *D. melanogaster* FKBP39, and we report for the first time the quaternary structure of the tetrameric arrangement of the NPL domain in the context of the full-length protein.

Based on resolved crystal structures of the N-terminal core domains of different nucleoplasmins widely described in the literature, the defining hallmark of this family is assumed to be an oligomeric β-propeller structure that consists of a set of five monomers that fit together to form a stable pentamer^40^,^41^,^42^,^43^. Recently, a domain sharing significant similarities with pentameric nucleoplasmin N-terminal core domains, termed NPL, was found on the N-termini of some nuclear proteins. For example, TgNF3 from *Toxoplasma gondii*, which associates with chromatin and binds certain gene promoters, consists of an N-terminal NPL domain followed by an acidic region and a divergent C-terminal domain of enigmatic function^44^. Plant HD-tuins have an N-terminal NPL domain accompanied by a C-terminal zinc finger. NPL domains have been found in some nuclear FKBPs, including *D. melanogaster* FKBP39 and *S. cerevisiae* Fpr4^18^. Current knowledge of the folding and structures of proteins with NPL domains is mostly based on studies of isolated NPL domains by small angle scattering of neutrons and X-rays or NMR, which revealed that the structure of isolated NPL resembles the structure of the pentameric nucleoplasmin core domain. Our results, however, demonstrate that the full-length protein may possess different structural organization. SAXS modelling of FKBP39 revealed that, in fact, oligomerization occurs via the NPL domain. However, in contrast to the isolated domain, which forms a pentamer, in the full-length FKBP39, NPL forms a tetramer. Indeed, NPLs form the core of the protein, and FKBDs are linked to it by intrinsically disordered, highly flexible chains, forming tentacle-like segments. Overall, the structure of the protein is very flexible and dynamic. Interestingly, full-length *D. melanogaster* CRP1 protein (also referred to as nucleoplasmin (dNLP)), for which the crystal structure of the isolated nucleoplasmin core domain was pentameric^41^, was shown to be predominantly tetrameric with a small fraction of pentamers in chemical crosslinking experiments^45^. This result clearly shows that, despite the sequence similarities between the nucleoplasmin core domains in the family, significant differences exist in their structural organization. Because NPL and nucleoplasmin core domains may behave differently when isolated compared to when they are a part of a longer polypeptide chain, our knowledge of the structural organization of full-length proteins possessing these domains requires reconsideration. We believe that this intriguing discrepancy between the structures of the isolated NPL domain and full-length protein may be related to the exposure of different or alternative oligomerization interfaces controlled by the distal part of the protein, i.e., by a long disordered linker alone or together with the C-terminal FKBP39 domain. In fact, the disordered distal region of the nucleoplasmin from *Xenopus laevis* was recently shown to promote the recognition of distinct oligomerization states of histones^46^. Thus, one cannot exclude the possibility that the flexible linker region of FKBP39 might be responsible for fine-tuning the tetramer structure and the oligomerization potential of the N-terminal NPL domain, similar to the protein-nucleosome interaction. To determine the functional relationships between any domains in the multi-domain protein, one needs a systematic understanding of the properties of each domain. To gain deeper insight into the structure of the full-length FKBP39 molecule and formation of alternative oligomeric states by NPL domain, future work will require detailed, domain-centric analyses to understand the molecular roles of individual regions. Such comprehensive analyses of the isolated regions seem to be vital for further elucidation of structural properties, geometry, interactions between the domains, and their self-association schemes. The putative role of the disordered FKBP39 linker in oligomerization and an in-depth analysis of the pentamer-to-tetramer transition when going from the isolated NPL to the domain in the full-length protein are also important for further studies of the probable mechanism of action of nuclear FKBPs, which have a NPL domain at their N-termini and are involved in chromatin structure regulation and gene expression.

Our SEC-MALS results revealed that some minor fractions of FKBP39 molecules existed in other quaternary structures (monomer and dimer; see the Results section). This finding suggests that FKBP39 possesses unique characteristics that enable it to form multiple complexes. Other types of oligomers may also be very important for its involvement in other nuclear regulatory and signalling functions. As shown by Li *et al*.^3^, FKBP39 is a key regulatory protein of a putative dynamic multiprotein complex that crosslinks hormonal signalling pathways. Indeed, two proteins, FKBP39 and Chd64, orchestrate the formation of this complex. Because FKBP39 is involved in the complex, its molecules must be able to switch conformation and, thereby, allow interaction with diverse partners, proteins and DNA. The extreme plasticity and flexibility of the FKBP39 molecule, which may exist in some conditions in an alternative quaternary structure, may lay a foundation for this multifunctionality. Currently, the exact function of FKBP39 is unknown; however, this protein was found to interact with histones^18^. Similarly, Fpr4, which also possesses an N-terminal NLP and C-terminal FKBD linked by a highly charged region, regulates chromatin structure via direct interaction with the H3-H4 tetramer^14^,^15^. According to Nelson *et al*.^15^, Fpr4 binds to H3 via its NPL, bringing its FKBD into close proximity to its substrate, P38, in a disordered tail of H3. Because FKBDs from FKBP39 possess all critical catalytic residues, this protein might also be involved in peptidyl-prolyl *cis-trans* isomerization. If FKBP39 existed in a pentameric arrangement, an intriguing question would arise: How do molecules with pentameric symmetry interact with nucleosomes with even-numbered subunits? Our findings clearly show that FKBP39 exists in an alternative tetrameric assembly that resembles a stochastic machine in which globular domains are attached to a common disordered region. In such molecular machines, flexible regions function by colocalization of the domains^47^. Therefore, the existence of four FKBDs in a tentacle-like structure would enable their rapid deposition in close proximity to their substrates and result in a local *increase* in their concentration. Their delivery together in a package may provide a focused response to existing signals, leading to synchronized reorganization of chromatin and changes in gene expression patterns.

Importantly, our findings show that FKBP39 concentrates in the nucleoli of COS-7 cells, consistent with previous observations by Edlich-Muth and coworkers in *Drosophila* cells^18^. Comparable subnuclear distributions of FKBP39 in different cell lines may indicate that its nucleolar localization signals and, probably, its function were strongly preserved during evolution. Interestingly, nucleolar Fpr4 was also shown to be involved in rDNA silencing^13^. Visible dots and small clusters of YFP-FKBP39 in the nuclear area might be associated with its putative chaperone activities occurring on the multiple levels of dynamic reorganization of chromatin structure. It is worth emphasizing that members of the FKBP family have been identified as histone chaperones^48^. However, the molecular basis of these activities of FKBP39 required further exploration.

One very important feature of *D. melanogaster* FKBP39 is its central highly charged region, which is formed by acidic and basic stretches located between the NPL and FKBD domains that, according to our findings, lack stable structures. The web-based predictors IUPred and PONDR-VLXT gave a high score for disorder tendency in that fragment, in agreement with SAXS and CD spectroscopy results revealing that 34.6 ± 0.2% of the polypeptide chain exists in a disordered conformation. The role of a disordered region as a functional platform surrounded by, or existing parallel to, a stable ordered structure in a proteinaceous molecule is well documented^21^. ID may also be a key factor of an assembly of supramolecular complexes^49^. ID has been reported to be important for the functioning of transcriptional machinery. Additionally, it is frequently involved in interactions between transcription factors and their partners (proteins and DNA)^50^. DNA binding usually occurs via basic domain/regions of various lengths. Li *et al*.^3^ showed that FKBP39 binds JHRE. Interestingly, in the basic region of FKBP39, bioinformatic analysis predicted the existence of a basic helix-loop-helix (bHLH) motif^51^, which is known for DNA binding^52^. Because analysing the CD spectra revealed that only 3.1 ± 0.3% of FKBP39 amino acid residues form α-helical structures, the bHLH motif is not likely to be present in FKBP39. Nevertheless, basic regions involved in DNA binding are likely to be disordered in isolation and presumably undergo a disorder-to-order transition upon binding^50^. In the basic region of FKBP39, PONDR-VLXT indicated short fragments that may correlate to MoRE. In isolation, these fragments possess a disordered conformation but have a high propensity for order^25^. Therefore, it is possible that FKBP39 binds to DNA via its disordered basic regions, which may undergo disorder-to-order transition and fold into a bHLH-like structure.

To summarize, *D. melanogaster* FKBP39 is a prototype protein with peculiar and very unique molecular characteristics. Our findings show that in contrast to current thought regarding the preserved pentameric structure of nucleoplasmins and proteins possessing an N-terminal NPL domain, the quaternary structure of the full-length FKBP39 is tetrameric. Because of the presence of flexible, tentacle-like disordered regions between NPL and FKBD, the tetramer of FKBP39 appears to be adjustable and very dynamic. Tetrameric molecular organization may be important for association with evenly numbered histones during nucleosome assembly and chromatin reorganization. Additionally, it may condition a rapid response to existing signals and tight regulation of gene expression and chromatin organization.

## Materials and Methods

### Chemicals

All buffers were prepared at 24 °C. The lysis buffer consisted of 20 mM Na_2_HPO_4_, 150 mM NaCl, 1 mM β-mercaptoethanol, and 0.2 mg/ml phenylmethylsulfonyl fluoride (PMSF), pH 7.0. Buffer A consisted of 50 mM Na_2_HPO_4_, 300 mM NaCl, and 1 mM β-mercaptoethanol, pH 7.0. Buffer B consisted of 50 mM Na_2_HPO_4_, 300 mM NaCl, 1 mM β-mercaptoethanol, and 250 mM imidazole, pH 7.0. Buffer H contained 50 mM Na_2_HPO_4_, 1000 mM NaCl, and 1 mM β-mercaptoethanol, pH 7.0. Buffer L consisted of 50 mM Na_2_HPO_4_ and 1 mM β-mercaptoethanol, pH 7.0. Buffer C contained 50 mM Na_2_HPO_4_ and 150 mM NaCl, pH 7.0.

### *In silico* analysis

The IDRs were analysed using PONDR-VLXT and Uversky plots (available at http://www.pondr.com^53^,^54^ and IUPred at http://iupred.enzim.hu)^22^,^55^. All analyses were performed using the default settings.

### Construction of expression vector

A cDNA clone encoding the full-length FKBP39 (LD30817) obtained from the *Drosophila* Genomics Resource Center (DGRC) was used as the polymerase chain reaction (PCR) template. The *Escherichia coli* strain XL1-Blue was used for all cloning procedures (Novagen). To amplify the full-length FKBP39 cDNA, the following forward and reverse primers were used for PCR amplification: 5′-ggggcg*ggatcc*TCGATGTTTTGGGGTTTGAAC-3′ (forward) and 5′-ccgggc*aagctt*ATGCACAGCTTTCAGTTCCAC-3′ (reverse). The forward and reverse primer sequences included the *Bam*HI and *Hin*dIII restriction sites, respectively. The uppercase letters in the sequences were derived from the FKBP39 gene, whereas the lowercase letters represent nucleotides added for cloning purposes; the restriction sites are shown in italics. After double-digestion with *Bam*HI and *Hind*III, the cDNA product was cloned into the corresponding sites of the modified pQE80L (Qiagen) vector pQE80L-XH, which added the 8× His-tag to the C-terminus (XH). The presence of the insert within the vector was confirmed by restriction analysis (data not shown), and the purified construct was verified by DNA sequencing.

### Expression and purification of FKBP39

First, 100 ml of Terrific Broth (TB) (Invitrogen) medium containing appropriate antibiotics (35 μg/ml chloramphenicol and 50 μg/ml carbenicillin) was inoculated with the pQE80L-XH-FKBP39-transformed *E. coli* strain BL21(DE3)pLysS (Novagen). After incubating overnight at 29 °C and 182 rpm, the starter culture was used to inoculate 4 l of TB medium with appropriate concentrations of antibiotics (as above). The culture was grown in 250 ml portions under the same conditions as the starting culture. When the OD_600_ of the growing culture reached a value of 0.6–0.8, production of the recombinant protein was induced by adding 0.25-mM isopropyl-β-D-thiogalactopyranoside (IPTG). After 3 h of incubation, bacterial cells were harvested by centrifugation for 10 min at 4,000 × g and 4 °C. Each cell pellet from 500 ml of the original culture was suspended in 12 ml of lysis buffer and frozen at −80 °C. The frozen cells from 1 l of culture were disrupted by thawing in a 25 °C water bath and freezing twice. As the second thawing began, the cells were supplemented with an appropriate volume of PMSF (0.2 mg/ml), β-mercaptoethanol (1 mM), DNase I (10 μg/ml) and RNase A (10 μg/ml). The lysates were incubated on ice until the nucleic acids were completely digested. The cell extract was subsequently clarified by centrifuging for 1 h at 17,500 × g and 4 °C. The supernatant was collected and supplemented with 0.2 mg/ml PMSF and purified using immobilized metal ion affinity chromatography. The cell lysate was incubated for 1 h at 4 °C and 40 rpm with 1,200 μl of Co^2+^-TALON resin (Clontech) that had been equilibrated previously with buffer A. Then, the resin was loaded onto a Tricorn 5/50 column (Amersham Biosciences) and washed 5 times with 5 ml of buffer A. The column was eluted at 0.5 ml/min on the ÄKTAexplorer (Amersham Biosciences) system at room temperature. We experimentally elaborated a purification protocol that includes six defined sub-steps during affinity chromatography. During the sub-steps, chromatography was performed until the curve of absorption at 280 nm plateaued. In the first and second sub-steps (ion-strength sub-steps), the resin was washed with buffer H and buffer L, respectively, to remove some of the contaminating proteins. Next, three imidazole gradient sub-steps were performed. In the third, fourth and fifth sub-steps, 25 mM, 75 mM and 100 mM imidazole, respectively, were used to wash out impurities that had bound non-specifically to the resin. Finally, the fusion protein was eluted with buffer B. The eluted FKBP39 was concentrated to a volume of 0.5 ml using an Amicon Ultra-4 Centrifugal Filter Unit (Millipore). The sample was then chromatographed over a Superdex 200 10/300 GL column equilibrated with buffer C. The column was eluted at room temperature at a flow rate of 0.5 ml/min on the ÄKTAexplorer (Amersham Biosciences) system. Fractions containing pure recombinant protein were collected, combined and stored at −80 °C. The concentration of purified protein was determined spectrophotometrically at 280 nm. The extinction coefficient of FKBP39 was calculated according to the method proposed by Gil and von Hippel^56^ as 0.598 ml/(mg × cm). The protein content and purity were evaluated after every stage of purification with SDS-PAGE^57^ and Coomassie Brilliant Blue R-250-stained gels^58^. The identification of the purified protein was performed by ESI-MS, as described previously^4^.

### CD spectroscopy

CD measurements were collected with a JASCO J-815 CD-spectropolarimeter equipped with a JASCO Peltier temperature controller (CDF-426S/15). The spectra were collected in both the far-ultraviolet (UV) region (190–260 nm) and near-UV (260–340 nm) region. Quartz cuvettes with path lengths of 1 mm (far-UV) and 10 mm (near-UV) were used. The FKBP39 concentration in buffer C was 10 μM and 25 μM for the far-UV and near-UV analyses, respectively. The final spectra were obtained by averaging five measurements performed at 20 °C with a scanning speed of 20 nm/min, a data resolution of 1.0 nm and a bandwidth of 1.0 nm. All spectra were corrected for the effect of the buffer, and all measurements were converted to molar residual ellipticity units^28^. The secondary structure content was calculated using CDPro spectra deconvolution software with IBasis 4 as the reference protein data set. The means and standard deviations were calculated for the results obtained using three algorithms: CDSSTR, SELCON3 and CONTINLL^59^.

### Analytical SEC

Analytical SEC was performed as described previously^60^. Briefly, a Superdex 200 10/300 GL column was equilibrated with buffer C and calibrated using a set of standard proteins^60^. The column volume (V_t_) was 24 ml, and the column void volume (V_0_) was determined to be 7.86 ml using blue dextran. The partition coefficients (K_av_) were calculated for each protein using the following equation: K_av_ = (V_e_ − V_0_)/(V_t_ − V_0_)^61^. The R_s_ values for each protein were plotted against the corresponding K_av_ to obtain a standard curve. Finally, 100 μl of the FKBP39 sample was chromatographed on the column at 1 mg/ml, and the V_e_ values were used to calculate its K_av_ and corresponding R_s_.

### Analytical ultracentrifugation

The AUC experiments were performed using a Beckman An-60 Ti rotor in a Beckman Optima XL-1 analytical ultracentrifuge. Samples were prepared in buffer C. SV-AUC experiments were conducted using standard double-sector charcoal-filled Epon^®^ centrepieces and quartz windows at 20 °C. Scans were recorded using absorbance optics. The measurements were made at 280 nm and 40,000 rpm for 0.2 and 0.5 mg/ml of protein. Multiple scans were fitted to a continuous size distribution using SEDFIT^29^,^62^. The partial specific volume of the FKBP39 (0.727 ml/g), solvent density (ρ = 1.00868 g/cm^3^) and viscosity (η = 0.010345 mPa × s), and Mm values of the monomer (40.8 kDa) and tetramer (162.8 kDa) were estimated using SEDNTERP^63^. SE-AUC analyses were performed using short solution columns (150 μl) at 4 °C with protein concentrations of 0.21, 0.44 and 0.96 mg/ml. The experiments were conducted at three different speeds: 6,500, 9,500 and 12,000 rpm. The scans were collected every 2 h in 10 replicates until equilibrium was reached, and the UV absorbance at 250 nm and 280 nm was recorded. The buffer density and viscosity and the sample partial specific volume were calculated as described above. The data were globally fitted to a single species in an interacting system model using SEDPHAT^64^.

### SEC-MALS

First, 100 μl of FKBP39 (1 mg/ml) was fractionated on a Superdex 200 Increase 10/300 column (GE Healthcare) equilibrated with buffer C at 0.5 ml/min. Elution of the FKBP39 complexes was monitored by the following in-line detectors: UV 280/254 nm (1260 Infinity LC, Agilent Technologies), light scattering (DAWN HELEOS II, Wyatt Technology) and differential refractometry (Optilab T-rEX, Wyatt Technology). Data analysis and Mm calculations were performed using ASTRA 6 software (Wyatt Technology).

### DLS

The R_s_ and %Pd values of FKBP39 were determined by DLS using a DynaPro NanoStar instrument (Wyatt Technology). The measurements were performed using disposable, 50 μl cuvettes (UVette, Eppendorf) for 0.25 mg/ml and 1.0 mg/ml of protein. The final result was averaged over 10 acquisitions, 5 seconds each. All calculations were performed using Dynamics 7 software (Wyatt Technology).

### SAXS

SAXS data for FKBP39 were collected using synchrotron radiation. The SAXS data set was recorded on the soft condensed matter beamline B21 at the Diamond Light Source (Harwell Science and Innovation Campus, Didcot, Oxfordshire, UK). Scattering data were collected within the s-vector range from 0.04 to 4 nm^−1^ using a PILATUS3 2 M hybrid photon-counting detector (Dectris, Switzerland). FKBP39 solutions (1.47 and 3 mg/ml in buffer C) were injected into the flow cell using the EMBL Arinax sample-handling robot. During 180 s of exposure to synchrotron radiation (λ = 0.1 nm), 18 independent SAXS patterns were recorded. The scattering data were processed using the JAVA-based package SCÅTTER (http://www.bioisis.net) and the PRIMUS program from the ATSAS package^65^. The Mm of the FKBP39 protein was calculated based on the SAXS data in the MoW program^30^. The structural parameters characterizing the FKBP39 molecule (e.g., R_g_ and p(r)) were estimated using the GNOM program in the ATSAS package^65^. The population of models of the FKBP39 tetramer was generated based on experimental data and calculated using the EOM package^37^,^38^. The tetramer of the NPL domain was generated using the SymmDock server^35^. EOM models were processed using the PD2 ca2main program (http://www.sbg.bio.ic.ac.uk/~phyre2/PD2_ca2main/).

### Analysis of the subcellular distribution of YFP-FKBP39

The full-length cDNA sequence of *D. melanogaster* FKBP39^66^ was amplified by PCR and fused to YFP by ligation with the pEYFP-C1 vector (Clontech) using *Hind*III and *EcoR*I restriction sites. YFP was attached at the N-terminus of FKBP39. The cDNA clone LD30817 described above was used as a template for PCR amplification. The recombinant plasmid construct was verified by sequencing. African green monkey kidney fibroblast COS-7 cells (ATCC CRL-1651) were transfected with 6 μg of DNA/300000 cells using 1 mg/ml polyethylenimine (PEI) solution (Sigma, Poland). The expression of the fusion protein (YFP-FKBP39) was confirmed by Western blotting using an anti-green fluorescent protein (GFP) antibody (Clontech). Confocal fluorescence microscopy images of fluorescently labelled FKBP39 were acquired using the Leica TCS SP5 II confocal system (Leica Microsystems GmbH, Germany) equipped with an argon laser and a 63xOil (NA: 1,4) objective lens.

## Additional Information

**How to cite this article**: Kozłowska, M. *et al*. Nucleoplasmin-like domain of FKBP39 from *Drosophila melanogaster* forms a tetramer with partly disordered tentacle-like C-terminal segments. *Sci. Rep.* **7**, 40405; doi: 10.1038/srep40405 (2017).

**Publisher's note:** Springer Nature remains neutral with regard to jurisdictional claims in published maps and institutional affiliations.

## Supplementary Material

Supplementary S1

## Figures and Tables

**Table 1:** Estimation of the FKBP39 secondary structure content from CD spectra.

α-Helix (%)			β-Strand (%)			Turns (%)	Other (%)
Regular	Distorted	Total	Regular	Distorted	Total	Turns	Unordered
0.2 ± 0.0	2.8 ± 0.4	3.1 ± 0.3	25.1 ± 0.3	14.2 ± 0.2	39.3 ± 0.5	22.8 ± 0.7	34.6 ± 0.2

The secondary structure content was calculated using CDPro spectra deconvolution software with IBasis 4, which is a reference set of 43 proteins. The means ± standard deviations were calculated for results obtained from three algorithms: CDSSTR, SELCON3 and CONTINLL.

**Table 2:** Molecular mass values determined by various independent techniques.

	Mm (monomer)^a^	Theor. tetramer	SV-AUC	SE-AUC	MALS	SAXS
Mm [kDa]	40696.6 ± 2	162.8	160.7	156.8	151.0	158.0 ± 14

^a^Determined by ESI-MS [Da].
