# Supplementary material for: Nucleoplasmin-like domain of FKBP39 from Drosophila melanogaster forms a tetramer with partly disordered tentacle-like C-terminal segments
Source: Sci Rep. 2017 Jan 11;7:40405. doi: 10.1038/srep40405 (PMC5225439; doi:10.1038/srep40405)
Supplement: Supplementary S1 [file srep40405-s1.pdf]

Nucleoplasmin-like domain of FKBP39 from *Drosophila melanogaster* forms a tetramer with partly disordered tentacle-like C-terminal segments

Małgorzata Kozłowska[1], Aneta Tarczewska[1], Michał Jakób[1], Dominika Bystranowska[1], Michał Taube[2], Maciej Kozak[2], Mariusz Czarnocki-Cieciura[3,4,5], Andrzej Dziembowski[4,5], Marek Orłowski[1], Katarzyna Tkocz[1] and Andrzej Ożyhar[1,*]

[1] Department of Biochemistry, Faculty of Chemistry, Wrocław University of Science and Technology, Wybrzeże Wyspiańskiego 27, 50-370 Wrocław, Poland

[2] Department of Macromolecular Physics, Faculty of Physics, Adam Mickiewicz University, Umultowska 85, 61-614 Poznań, Poland

[3] International Institute of Molecular and Cell Biology in Warsaw, 02-109 Warszawa, Poland

[4] Institute of Biochemistry and Biophysics, Polish Academy of Sciences, 02-106 Warszawa, Poland

[5] Department of Genetics and Biotechnology, Faculty of Biology, University of Warsaw, 02-106 Warszawa, Poland

* To whom correspondence should be addressed. Tel: 0048 71 320 63 33; Fax: 0048 71 320 63 37; Email: andrzej.ozyhar@pwr.edu.pl

The authors wish it to be known that, in their opinion, the first 2 authors should be regarded as joint First Authors

Analytical size-exclusion chromatography (SEC)

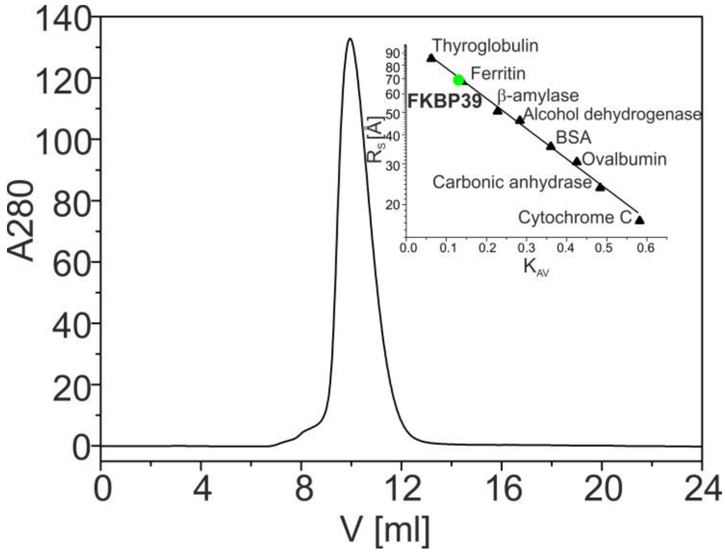

Figure S1. Analytical SEC of FKBP39. The protein was chromatographed on a Superdex 200 10/300 GL column equilibrated with buffer C at room temperature and a flow rate of 0.5 ml/min; an injection volume of 0.1 ml was used, and the protein concentration was 1 mg/ml. The inset shows the calibration curve determined using standard proteins (black triangles). The grey dot corresponds to FKBP39.

During SEC under native conditions, FKBP39 was eluted from the column with an elution volume (V_e) corresponding to a surprisingly large Stokes radius (R_s) of 69 Å, which falls near to the R_s value of the relatively large globular protein ferritin (450 kDa) (inset). The theoretical R_s for a globular protein with a Mm similar to that of FKBP39 is 27.8 Å, indicating that the experimental R_s is 2.5 times larger than the theoretical value. Similarly, experimental and theoretical values of the molecular volume and density were also substantially different (Table S1). The determined apparent Mm of 419±5 kDa was more than 10 times larger than the value determined by ESI MS (40.696 kDa).

Summary of hydrodynamic properties determined by analytical SEC

Table S1. Hydrodynamic properties of FKBP39.

	R_s [Å]		$V_s \times 10^3$ [Å^3]		ρ [Da/Å^3]	
Mm [kDa]	Theor.[a]	Exp.	Theor.[b]	Exp.[c]	Theor.[b]	Exp.[c]
40.695	27.8	69.4±0.3	90.2	1400.1	0.45	0.03

[a]Determined from the equation: $\log (R_s^N) = (0.819 \pm 0.025) + (0.388 \pm 0.012) \times \log (Mm)$ for native globular proteins. [b]Calculated using theoretical R_s. [c]Calculated using experimentally determined R_s.

Dynamic Light Scattering (DLS)

FKBP39 at 1.0 mg/ml

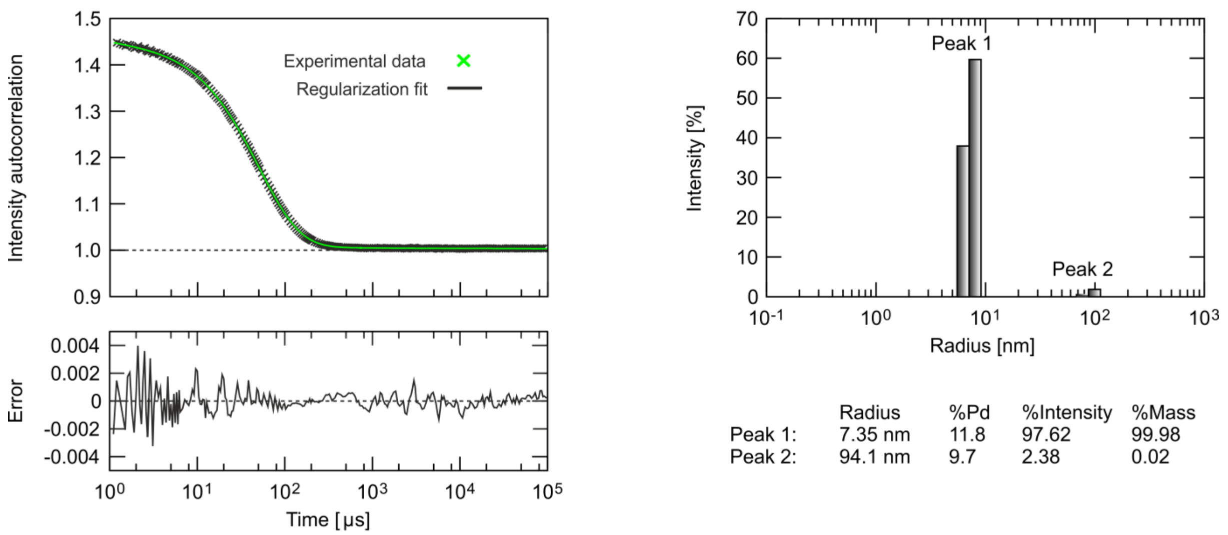

	Radius	%Pd	%Intensity	%Mass
Peak 1:	7.35 nm	11.8	97.62	99.98
Peak 2:	94.1 nm	9.7	2.38	0.02

FKBP39 at 0.25 mg/ml

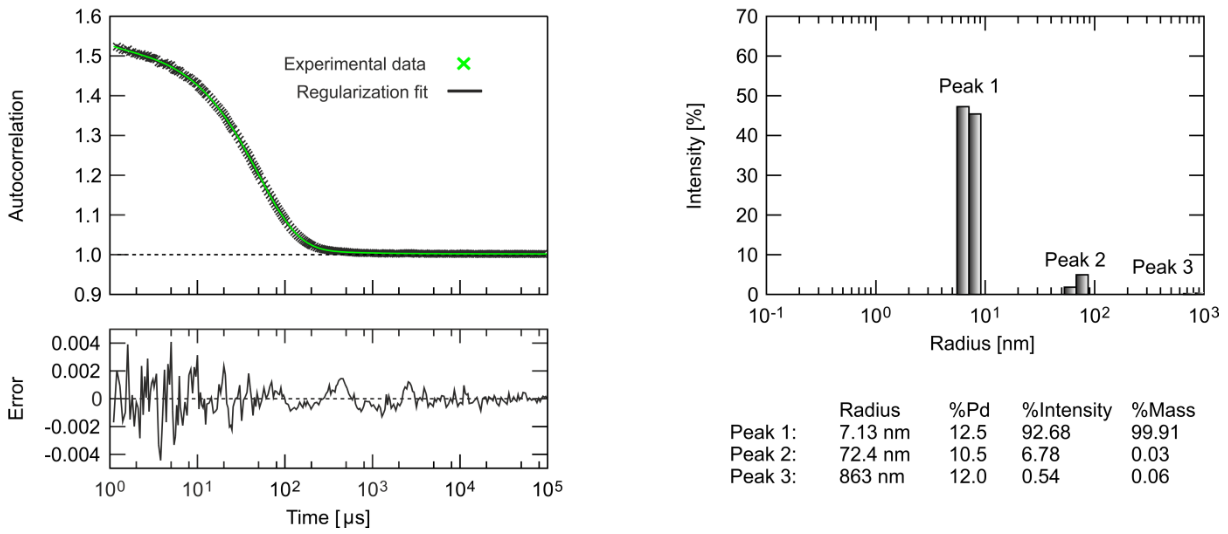

	Radius	%Pd	%Intensity	%Mass
Peak 1:	7.13 nm	12.5	92.68	99.91
Peak 2:	72.4 nm	10.5	6.78	0.03
Peak 3:	863 nm	12.0	0.54	0.06

Figure S2. Particle size analysis by DLS. R_s and %Pd were calculated based on regularization fit. %Intensity corresponds to percent of signal intensity assigned to different peaks whereas %Mass to percent of total sample mass assigned to different peaks calculated using Rayleigh Spheres model.
